# Using Constrained Density Functional Theory to Track Proton Transfers and to Sample Their Associated Free Energy Surface

**DOI:** 10.1021/acs.jctc.1c00609

**Published:** 2021-09-01

**Authors:** Chenghan Li, Gregory A. Voth

**Affiliations:** Department of Chemistry, Chicago Center for Theoretical Chemistry, James Franck Institute, and Institute for Biophysical Dynamics, University of Chicago, Chicago, Illinois 60637, United States

## Abstract

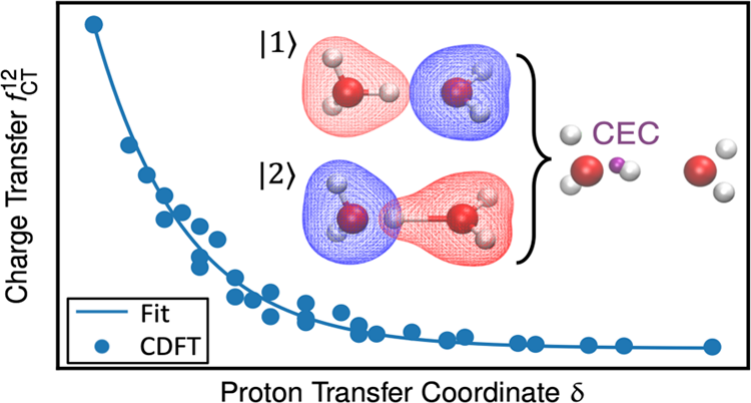

Ab initio molecular dynamics (AIMD) and quantum mechanics/molecular mechanics (QM/MM) methods are powerful tools for studying proton solvation, transfer, and transport processes in various environments. However, due to the high computational cost of such methods, achieving sufficient sampling of rare events involving excess proton motion—especially when Grotthuss proton shuttling is involved—usually requires enhanced free energy sampling methods to obtain informative results. Moreover, an appropriate collective variable (CV) that describes the effective position of the net positive charge defect associated with an excess proton is essential both for tracking the trajectory of the defect and for the free energy sampling of the processes associated with the resulting proton transfer and transport. In this work, such a CV is derived from first principles using constrained density functional theory (CDFT). This CV is applicable to a broad array of proton transport and transfer processes as studied via AIMD and QM/MM simulations.

## Introduction

The accurate and efficient delineation of proton transport (PT) and its associated mechanism continues to be fundamentally important in chemistry, biology, and materials science.^1−3^ Excess proton transport in aqueous and biomolecular environments involves rearranging covalent and hydrogen bonds, which is known as the Grotthuss hopping or shuttle mechanism.^4,5^ Due to this chemically reactive nature of the process, the ab initio molecular dynamics (AIMD) method,^6,7^ which treats the electronic degrees of freedom explicitly and “on the fly” along with the dynamics of the nuclei, provides one popular approach for modeling PT at an atomistic level. Among the various possible electronic structure methods, density functional theory (DFT) represents a powerful approach for implementing AIMD, as DFT has a reasonable balance between accuracy and computational efficiency.

However, even when using the generalized gradient approximation (GGA) level of DFT,^8^ the high computational cost of AIMD typically limits the sampling of the MD to within the subnanosecond timescale for systems containing several hundreds of electrons. This limitation can prevent an adequate sampling of rare events, such as PT involving weak acids that are commonly found as protonatable amino acids in protein channels and transporters. In such cases, the high reaction barrier for proton dissociation from weak acids results in timescales that usually exceed nanoseconds; thus, enhanced free energy sampling methods to bias the PT process are necessary to obtain statistically and physically meaningful results.

Common enhanced sampling methods, such as umbrella sampling,^9^ metadynamics,^10^ and adaptive biasing force,^11^ add bias to one or more collective variables (CVs) to accelerate the sampling along these degrees of freedom. In the context of PT, identifying an appropriate CV that represents the position of the net positive charge defect associated with the excess proton is important. Due to frequent bond breaking and forming events in Grotthuss proton shuttling, the identity of the charge carrier species (hydronium-like or protonated weak acids) is dynamically changing, and the excess protonic charge defect tends to be distributed among several solvation shells instead of localizing on a central hydronium structure or on a weak acid. As such, a CV cannot be associated with any specific “proton” in the system but is more appropriately assigned in some way to be the charge defect associated with the excess proton, often referred to as the “center of excess charge” (CEC). For AIMD simulations, there are several CEC definitions that have been proposed, namely, mCEC,^12^ the proton indicator,^13^ and the more recent rCEC.^14^ However, a more rigorous definition based on ab initio theory is preferred. In this work, we present a variant of the CEC definition derived from a diabatic electronic structure method, the constrained DFT (CDFT),^15^ and apply it to two case studies: an excess proton in water and glutamic acid in water. We conclude by unraveling the collective motions encoded in the CEC via computing its IR spectrum as well as illustrating its ability to accelerate the sampling of proton transfer when combined with metadynamics.

## Theory of Constrained DFT

The CDFT framework was proposed for solving the electronic structure of a system subject to the following constraint on electron density
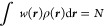
1where *w*(***r***) is the weighting function that defines the constraint and *N* is the constraint target value. The constrained lowest-energy state can be obtained from an optimization problem via the standard method of Lagrange multipliers
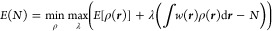
2Herein, *E*[*ρ*(***r***)] is the density functional, which in this work is BLYP^16,17^ and ωB97X,^18^ and *λ* is the Lagrange multiplier. The electron density determined from eq 2 thus deviates from the constraint-free adiabatic ground-state density, making it a so-called diabatic state. As molecules in the condensed phase sample more compact geometries on average, the promolecule formalism approach^15,19^ was employed and the system was partitioned into two molecular fragments, A and B. The constraint target value in eq 2 was then calculated from the total promolecule density by summing up the ground-state electronic density of the two fragments as if they were independent
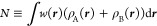
3Here, the Becke population^20^ scheme was used to define the weighting function as
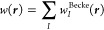
4where the summation index *I* refers to the atoms of protonated species in each diabatic state, i.e., a hydronium or a neutral glutamate. The expected behavior of the promolecule constraint is that the resulting diabatic electronic density will resemble as much as possible the superposition of two pure fragments, such as, e.g., a pure water and a pure hydronium in the case of the Zundel cation H_5_O_2_^+^.

The coupling between two diabatic states is calculated from the integral using the Kohn–Sham determinant |Φ_*i*_⟩^15,21^
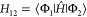
5A 2 × 2 Hamiltonian can be constructed using the diabatic energies from eq 2 as diagonal terms
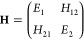
6Similarly, the overlap matrix is defined as
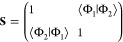
7Then, the so-called CDFT configurational interaction (CI)^15,19^ can be performed by solving the generalized eigenvalue problem
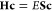
8The resulting eigenfunctions, ***c*** = {*c*_1_,*c*_2_}, determine the degree to which each of the two diabatic states contributes to the CI ground state of the system; we will use the ***c*** vector to define our CEC, as described in the next section.

## Theory of Center of Excess Charge

**Figure 1:**
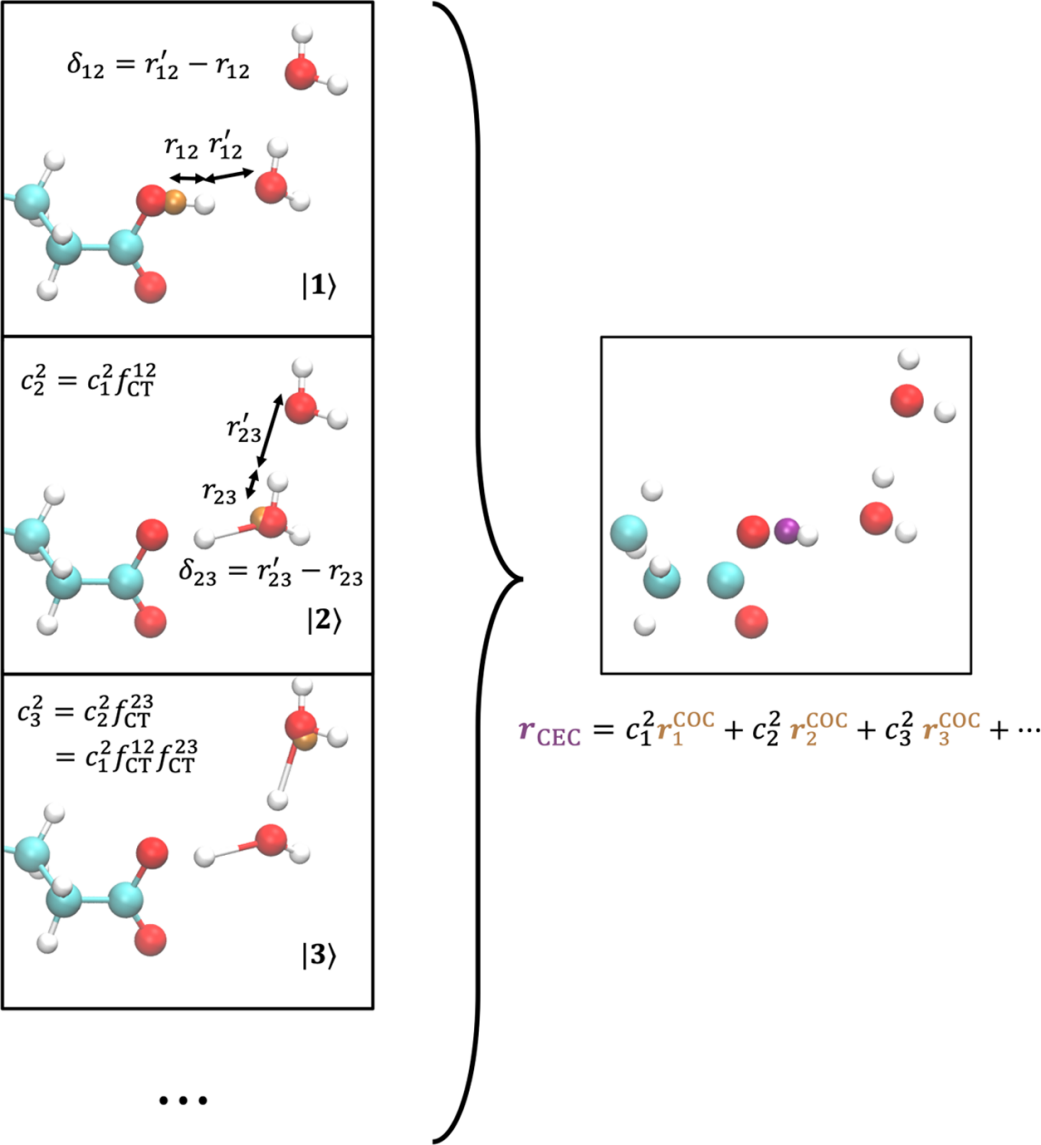
Illustration of CEC calculation for Glu in water. For the sake of clarity, only three diabatic states are shown, even though 20–30 states (on average) will be resolved in the condensed phase by searching up to three solvation shells of the hydrated excess proton. The COC in each state is rendered by an orange sphere. The resulting CEC as a linear combination of COCs is rendered in purple in the right panel. The *f*_CT_^12^ and *f*_CT_^23^ are computed using eq 12 as a function of δ_12_ and δ_23_, respectively.

Assuming that each diabatic state defines a bonding topology, e.g., in Figure 1, state |1⟩ defines a neutral glutamate and two neutral water molecules, while state |2⟩ defines a hydronium, a charged Glu, and a neutral water molecule as two of the possible topologies. Given the bonding topology of every diabatic state, the “diabatic” CEC within state |*i*⟩ is simply the center of charge (COC) of the species that carries protonic charges, i.e., the hydronium or protonated weak acid in |*i*⟩ in Figure 1, such that
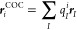
9Here, we assume that the diabatic excess charges are associated with atomic positions ***r***_*I*_ and are modeled by fixed charges *q*_*I*_^*i*^, the charge of atom *I* in state |*i*⟩. The fixed charge values are taken in this example from the CHARMM 36 force field^22^ and a prior paper^23^ and are also summarized in Table S1. In diabatic states with well-defined bonding topologies, the fixed force field charges are considered to be a reasonable description of the system, while the excess charge delocalization and the polarization due to the excess proton are characterized by the ***c*** vector. Since the coefficient vector ***c*** obtained from eq 8 represents the population of each diabatic state in the final CI ground state, the “adiabatic” CEC is naturally defined as the weighted average of each diabatic CEC (i.e., COC)
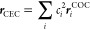
10In this sense, the *c*_*i*_’s measure the extent of excess charge transfer. Accordingly, we define the charge-transfer factor in what follows to represent the excess charge distribution between state |*i*⟩ and state |*j*⟩
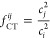
11

Due to the extended searching space introduced by the multiplier λ, the CDFT calculation (eq 2) is typically more expensive in comparison to the adiabatic electronic structure method used in AIMD. Therefore, we adopted an approximation of the ground-state vector ***c*** to compute the CEC on the fly in the AIMD simulations. It was found that an exponential function of proton transfer coordinate δ can provide a good fit for the charge-transfer factor
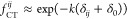
12The *δ*_*ij*_ is defined here as the difference between two O–H distances
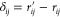
13where *r*_*ij*_ denotes the distance between the shared proton and the proton donor oxygen in state |*i*⟩ and *r*_*ij*_ denotes the distance between the proton and the proton acceptor in state |*j*⟩. The parameters *k* and *δ*_0_ were calibrated to match the exact *f*_CT_*ij* from CDFT-CI calculations between protonated species and water in the gas phase using BLYP or ωB97X functional, in this case by a least-squares fitting. The training configurations used are described in the Simulation Details section and provided as Supporting Information. The list of fitting parameters for the CEC is provided in Table 1. Further details pertinent to the parametrization procedure can be found in the Simulation Details section.

**Table 1:** Fitted Parameters of the CEC for Water and Glutamic Acid

	CDFT functional	*k* (Å^–1^)	δ_0_ (Å)
H_3_O^+^–H_2_O	BLYP	4.234	0
	ωB97X	4.898	0
Glu–H_2_O	BLYP	2.946	0.5361

To generalize the CEC to the condensed phase environment, we assume that the solvating waters of the hydronium or protonated acid propagate the excess charge to further solvation shells following the same exponential rule (eq 12) as illustrated in Figure 1. The diabatic states for the CEC calculation were selected by searching three solvation shells of hydrogen bond acceptors using a 2.5 Å criterion for the O–H distance, as described in more detail in ref (24). The further solvation shells were found to be not needed because the computed *c*_*i*_ values already diminished for the fourth shell. After resolving all of the charge-transfer factors between each proton donor–acceptor pair, the approximated *c*_*i*_^2^ was then computed from *f*_CT_^*ij*^ by applying the normalization condition (∑_*i*_*c*_*i*_^2^ = 1)
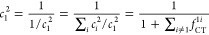
14a
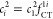
14bThe *c*_1_^2^ was computed directly from the charge-transfer factors (third equal sign in eq 14a), while other *c*_*i*_’s were available via eq 14b. In summary, eqs 9 and 10 define the CEC, while eqs 12, 14a, and 14b yield an approximation to the exact CDFT-CI ***c***, thereby facilitating CEC calculations at a reasonable computational cost.

Another way of viewing the CEC is the dipole moment of excess charges. Following eqs 9 and 10, we have
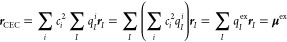
15where we define the excess charge of atom *I* as its weighted average charge ∑_*i*_*c*_*i*_^2^*q*_*I*_^*i*^. Hence, the excess charge contribution to the IR spectrum can be calculated directly from the CEC velocity correlation function
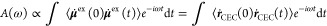
16

## Simulation Details

**Figure 2:**
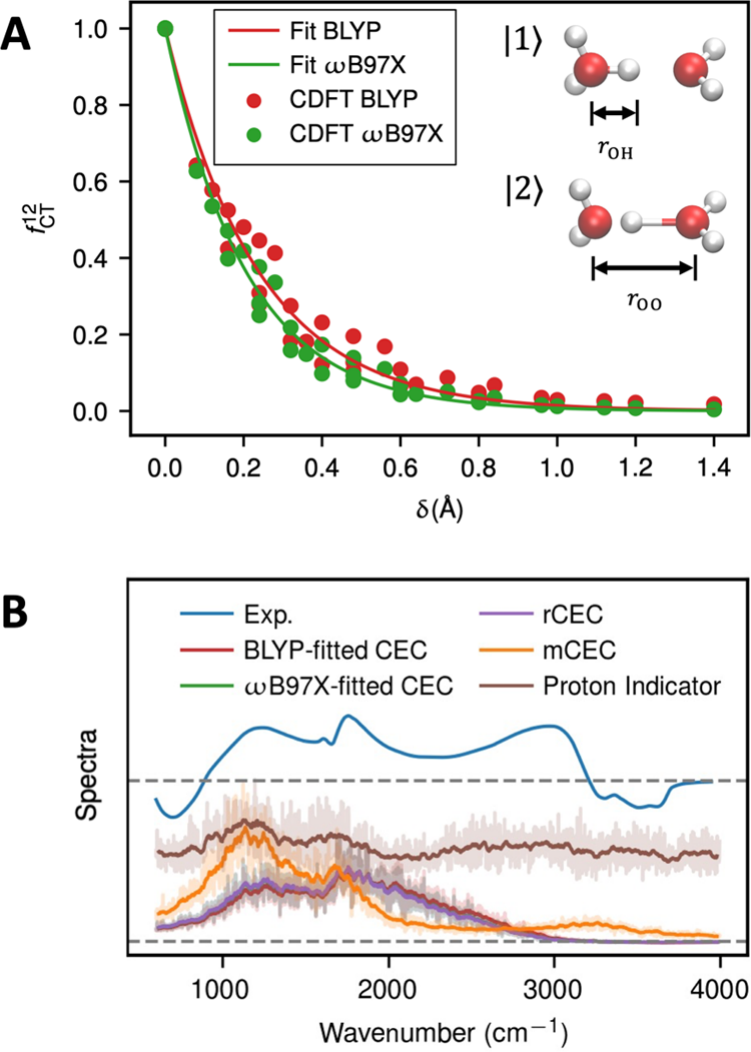
(A) Charge-transfer factor between hydronium and water calculated by CDFT with BLYP and ωB97X functionals and fitted curves. (B) Calculated AIMD excess charge spectrum of a hydrated excess proton in water using various CEC definitions. The experimental IR spectrum is the acid solution spectrum subtracted by the pure water spectrum, taken from ref (40). The computed CEC spectra are shown in transparent, while the running averages performed using a 33 cm^–1^ window are shown in solid colors. The proton indicator spectrum intensity was scaled by 0.5 for better presentation. Note that the ωB97X-fitted CEC (green) overlaps with the rCEC curve (purple), which very nearly overlaps with the BLYP-fitted CEC (red).

**Figure 3:**
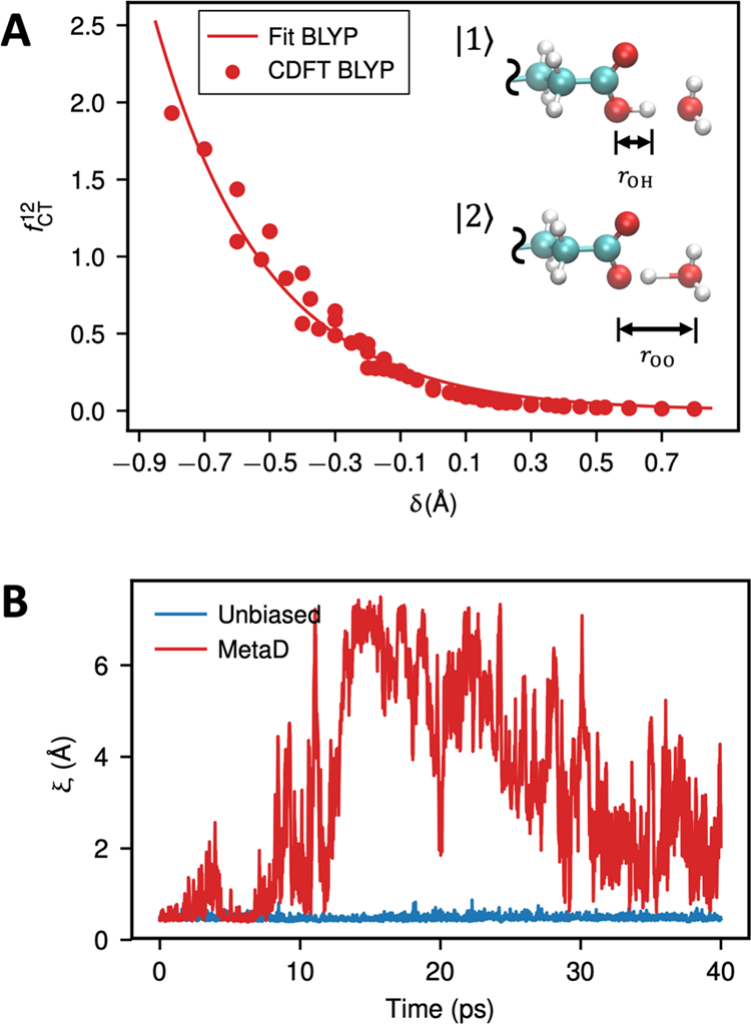
(A) Charge-transfer factor between glutamate and water calculated by CDFT with the BLYP functional and its fitted curve. (B) Time series of the proton disassociation CV *ξ* in an unbiased AIMD (blue) and a metadynamics run (red) of glutamate solution.

The CDFT calculations were conducted for hydronium–water (Figure 2A) and glutamate–water (Figure 3A); the electronic structure settings were identical to those used for AIMD simulations, as shown below. Both the ωB97X and the BLYP functionals were used for hydronium–water CDFT calculations, while the latter was adopted for glutamate–water. For hydronium–water, the total promolecule density of states |1⟩ and |2⟩ was calculated by adding the ground-state density of the hydronium defined in that state plus the water density, i.e., the hydronium and water were chosen to be the fragments A and B, respectively, in eq 3. For Glu–water, the promolecule density of state |1⟩ is the sum of neutral Glu density plus the neutral water density, and for state |2⟩, it is the sum of deprotonated Glu plus the hydronium. Established literature values^25^ for the atomic radii, including 0.75 Å for carbon, 0.32 Å for hydrogen, 0.63 Å for oxygen, and 0.71 Å for nitrogen, were used for calculating the Becke population. The fitting procedure involving the hydronium–water molecular pair was based on a training set comprised of a series of fixed values for the oxygen–oxygen distance, *r*_OO_, including 2.2, 2.4, 2.6, 2.8, 3.0, and 3.2 Å. For each value of *r*_OO_ in this set, 6 shared proton positions were sampled evenly from *r*_OH_ = 0.9 Å to *r*_OH_ = *r*_OO_/2, resulting in 6 × 6 = 36 data points. The training set for glutamate–water consists of 7 oxygen–oxygen distances evenly distributed from 2.2 to 2.8 Å and 9 oxygen–hydrogen distance values ranging from 1.0 Å to *r*_OO_-1.0 Å, resulting in 7 × 9 = 63 data points. The CDFT calculations were performed by the CDFT implementation^26^ in CP2K^27,28^ combined with Libxc.^29^ In CDFT calculations, the setup for the DFT part follows the same as for the AIMD described below. The convergence criterion for the CDFT was chosen to be 10^–3^ au.

The AIMD simulation of the excess proton in water was performed for 128 water molecules and 1 excess proton in a 15.64 Å × 15.64 Å × 15.64 Å box. The electronic structure was described by the BLYP-D3 density functional^30^ with Goedecker–Teter–Hutter pseudopotentials.^31^ The Gaussian and plane waves (GPW) method^32^ was used, the Kohn–Sham orbitals were expanded using the Gaussian basis set TZV2P, and the electronic density was expanded in plane waves with a cutoff of 400 Ry. The orbital transformation (OT) method^33,34^ with the direct inversion in the iterative subspace (DIIS) minimizer was used as the self-consistent field (SCF) method with a convergence criterion of 10^–6^ au. An experiment directed simulation (EDS) correction^35^ was also employed as a minimal add-on bias to correct the overly strong hydrogen bonding in most DFT functionals. It was found that excess proton and water diffusion better match experimental values after including the EDS correction. This EDS method for excess protons in water, which follows earlier work for pure water,^36^ is briefly summarized in the Supporting Information, and more details will be published in the future.

The system was first equilibrated in the constant NVT ensemble at 298 K for 30 ps, and then it was switched to the constant NVE ensemble for 200 ps for collecting non-thermostatted dynamical data. A timestep of 0.5 fs was used to integrate the system MD. All of the AIMD simulations were carried out with the CP2K program package, coupled with a modified version of PLUMED2^37^ for the EDS correction. Both the BLYP-fitted and the ωB97X-fitted CEC parameters were employed in the analysis of AIMD of proton in water.

The Glu–water system consisted of 1 neutral glutamate with 110 water molecules in a 16 Å × 16 Å × 16 Å box. We set up the electronic structure calculation similar to that used for the proton–water system, except that no EDS correction was used. The unbiased and well-tempered metadynamics^38^ simulations were conducted with the constant NVT ensemble at 300 K in CP2K with PLUMED2 for computing and biasing the CEC. To be consistent with the underlying density functional used to perform AIMD simulations, the BLYP-fitted hydronium–water and glutamate–water CEC parameters were used. The collective variable used in metadynamics was the minimum distance between the CEC and the two Glu carboxyl oxygens:
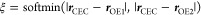
17where softmin is a smooth version of minimum function
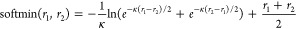
18where *κ* = 40 Å^–1^. The Gaussians in metadynamics were deposited every 50 fs with an initial height of 0.2 kcal/mol and a width of 0.1 Å. The bias factor of γ = 12 was used to account for a roughly 9 kcal/mol proton dissociation barrier.

## Results

The CDFT-calculated charge-transfer factors *f*_CT_^12^ and the fitted curves using eq 12 are shown in Figure 2A. The ωB97X-based value of the charge-transfer factor was found to decay more quickly compared to that calculated using the BLYP functional. This finding is not surprising because the range-separated hybrid functional ωB97X produces less charge delocalization in comparison to the GGA BLYP functional. However, for both cases, the exponential function (eq 12) provides a good fit. Given that *c*_1_^2^ = *c*_2_^2^ when the proton is equally shared between two water molecules, the *δ*_0_ parameter was set to be zero. Interestingly, the fitted parameter *k* from ωB97X was found to share a similar value with the one used in the rCEC parameters^14^ (4.898 Å^–1^ vs 4.984 Å^–1^), which is based on the multistate empirical valence bond method (MS-EVB),^23,24,39^ which better justifies the use of the rCEC variable for AIMD simulations.

As shown in an earlier study,^14^ the IR spectrum of the excess proton charge provides a systematic measure of the CEC dynamics by revealing the encoded collective motions in that CV. Due to the similarity in their parameters, we found that the CDFT-CECs in general align with the rCEC spectrum (Figure 2B), again justifying the use of the MS-EVB-derived CEC in AIMD simulations.^14^ It should be noted that although the two functionals exhibited different charge-transfer behaviors (Figure 2A), the BLYP-fitted CEC and the ωB97X-fitted CEC produced very similar spectra, as shown in Figure 2B; this correlation implies that the CEC parametrized by one functional may be applied to AIMD simulations using other functionals. Compared to the experimental IR difference spectrum, the CEC spectra reproduce the acid continuum 600–3200 cm^–1^, which is the signature feature of the acid solutions that arise from the hydrated excess proton. In particular, the proton transfer mode (PTM) at around 1200 cm^–1^ and the flanking water bending at around 1750 cm^–1^ are effectively reproduced in the excess proton CEC spectra, indicating that these modes are well reflected in the encoded collective motions of CEC. It should be noted that the CEC spectrum decays in the range of 2300–3200 cm^–1^, as opposed to the peak present in the experimental spectrum. This difference is due to the decaying excess proton charge in outer solvation shells, thereby reducing the intensity of this region, which is associated with the red-shifted O–H stretching in the second and third solvation shells of the excess proton, as detailed in a prior paper.^14^ Importantly, the CEC spectrum decays to zero at the same position as the experimental difference spectrum at around 3200 cm^–1^, revealing that the CEC excludes any bulk-like water O–H stretching in its motions. In summary, the CEC mostly represents the inner core motions of the protonated water complex, including the PTM and the flanking water bending, smoothly scales down its weight in the outer solvation shells, and is completely shut off for the bulk-like waters, which is the ideal behavior of a CV to focus only on PT and its related collective motions different from bulk-water fluctuations.

By contrast, the mCEC exhibits a nondiminishing intensity in the pure water region (3200–4000 cm^–1^), revealing its sensitivity to the bulk-water motions. This is an unfavorable behavior as illustrated previously^14^ in that the use of mCEC in enhanced sampling will bias the bulk-like water motions and may result in artificial water autoionizations, distant from the excess proton reaction center. The proton indicator, on the other hand, has nonzero absorption over the full frequency range, as a result of its discontinuity.^14^ This nondifferentiability prevents its application in MD-based enhanced free energy sampling simulations due to an energy conservation problem when biasing such a CV, which can lead to a violation of detailed balance.^41^

We then studied glutamate in water to examine the CEC as a collective variable to be used in enhanced free energy sampling. Accordingly, we first parametrized the CEC from CDFT calculations of charge transfer between glutamate and water. The *δ*_0_ parameter in eq 12 is needed for this case to account for the asymmetry in the PT between Glu and water. Figure 3A shows that the exponential function indeed outlines the charge-transfer behavior for Glu–water, suggesting that this functional form can be applied to PT involving other weak acids. We note that other forms of switching functions could also be employed if eq 12 is deficient in providing an accurate fit to the CDFT behavior for a particular system.

The proton disassociation barrier for Glu is large,^42^ given the experimental p*K*_a_ of around 4.2. Thus, dissociation tends to be a rare event compared to the achievable timescale of AIMD simulations. In Figure 3B, we show the sampling efficiency gained from a metadynamics run using the CV ξ, representing the distance between the CEC and the closest carboxyl oxygen (see Simulation Details for the definition). During the 40 ps AIMD run, the unbiased simulation was found to sample only the free energy well corresponding to a protonated Glu, while the metadynamics drives the CEC to easily sample the dissociation of the proton from Glu and almost completes a “round trip”.

## Conclusions

In this work, we developed an appropriate collective variable (CV) to define the charge defect location and transfer properties for proton transfer and transport processes, resulting in a more rigorous ab initio definition of the center of excess charge. We also showed that the charge-transfer behavior of CDFT can be approximated by an exponential function. We further examined the encoded collective motions in this newly defined CEC via calculating its IR spectrum. The full acid continuum was reproduced, suggesting the ability of this new CEC CV for capturing the excess proton motions without any contamination from other irrelevant degrees of freedom.

We also simulated a glutamate–water solution as an example, illustrating the use of the new CEC in enhanced free energy sampling for amino acid ionization in water. An AIMD metadynamics run driving the CEC was found to explore a much larger CV space than an unbiased AIMD run, providing efficient sampling of the proton disassociation of Glu.

We note that the present CDFT formalism is suitable for describing any charge-transfer reaction in which the bonding topology changes between the diabatic states; thus, it is not limited to the proton transfer processes studied herein. Therefore, the method can be generalized to identify appropriate CVs for other charge-transfer reactions, e.g., ATP or GTP hydrolysis.

## Supporting Information Available

Description of the experiment directed simulation (EDS) correction for hydrated excess proton AIMD simulations; table of atomic charges used to compute the center of charge (COC) (PDF)
Coordinate file used for fitting CEC parameters for water–water (XYZ)
Coordinate file used for fitting CEC parameters for glutamate–water (XYZ)

## Supplementary Material

ct1c00609_si_001.pdf

ct1c00609_si_002.xyz

ct1c00609_si_003.xyz
