# Supplementary material for: Using Constrained Density Functional Theory to Track Proton Transfers and to Sample Their Associated Free Energy Surface
Source: J Chem Theory Comput. 2021 Sep 1;17(9):5759–65. doi: 10.1021/acs.jctc.1c00609 (PMC8444337; doi:10.1021/acs.jctc.1c00609)
Supplement: Supplementary file 1 — ct1c00609_si_001.pdf [file ct1c00609_si_001.pdf]

Supporting Information for

Using Constrained Density Functional Theory to Track Proton Transfers and to Sample Their Associated Free Energy Surface

*Chenghan Li and Gregory A. Voth**

Department of Chemistry, Chicago Center for Theoretical Chemistry, James Franck Institute, and Institute for Biophysical Dynamics, University of Chicago, Chicago, IL, 60637

*Corresponding author: gavoth@uchicago.edu

EDS(OH) METHOD FOR HYDRATED EXCESS PROTON IN WATER

We extended the EDS(OH) correction originally developed for AIMD simulation of water[1] to simulate hydrated excess protons. In EDS(OH) water simulations, a bias potential (eq 1 in ref[1]) was added to all the O-H pairs in the system except for the pairs whose O-H distances are within 1.2 Å. The motivation was to correct the overly strong hydrogen bonds in GGA-level of DFT but not to disrupt the bonding structure of individual water molecules. This approach is applicable to nonreactive water simulations, but may be inadequate after introducing an excess proton into the system, since the proton transport via Grotthuss shuttling frequently samples proximate O-H contacts. Hence, it requires a smooth switch-on and -off of the EDS bias between O-H pairs during bond breaking and formation in the Grotthuss process to apply the EDS(OH) potential in simulating proton in water. Similar to the idea of using CDFT to define the CEC presented in this work, CDFT was employed to define diabatic states, each of which corresponds to a bonding topology in which the excess proton is regarded attached to a distinct water molecule. The EDS(OH) correction is then computed in each diabatic state only between nonbonded O-H pairs,

and the final EDS force, computed as a weighted average of the state-dependent EDS bias force, $\boldsymbol{F}^{\mathrm{EDS}} = \sum_i c_i^2 \boldsymbol{F}_i^{\mathrm{EDS}}$, is added to the DFT force. The c_i is computed using the same approximations in this work (eqs 14a and 14b in the main text).

Table S1. Atomic charges used to compute center of charge.

Excess proton carrier molecule	Atom	$q\ (e)$
H_3O^+	O	0.196
	H	0.268
Protonated Glu	O	0.581
	H	0.419

The O atom in Glu refers to the carboxylic hydroxyl oxygen and the H atom refers to the hydroxyl hydrogen, and other atoms in Glu are zero.

Supplemental References

1. Calio, P. B.; Hocky, G. M.; Voth, G. A., Minimal Experimental Bias on the Hydrogen Bond Greatly Improves Ab Initio Molecular Dynamics Simulations of Water. *J. Chem. Theory Comput.* **2020,** *16* (9), 5675-5684.
